# Consumers’ Psychology Regarding Attachment to Social Media and Usage Frequency: A Mediated-Moderated Model

**DOI:** 10.3390/bs14080676

**Published:** 2024-08-05

**Authors:** Cem Karayalçın, Eyyup Yaraş

**Affiliations:** 1 Department of Business Administration, Antalya Bilim University, 07190 Antalya, Turkey; 2 Department of Business Administration, Akdeniz University, 07058 Antalya, Turkey; eyaras@akdeniz.edu.tr

**Keywords:** social media marketing, brand equity, purchase intentions, consumer psychology, online consumer behavior, moderation analysis, mediation analysis

## Abstract

Although there are studies in the literature investigating the effect of social media marketing activities (SMMAs) on brand equity and purchase intentions, scant information is available regarding consumers’ attachment to social media (ASM) and usage frequency considering the abovementioned variables. Accordingly, one of the main purposes of this study is to investigate the effect of consumers’ ASM on their perceptions of SMMAs. The second main purpose of this study is to test whether social media usage frequency has a moderating effect regarding the impact of perceived SMMAs on brand equity and purchase intentions. Questionnaires were collected face-to-face and a data set of 907 Turkish youth consumers were evaluated. Two major international technology brands were selected for the purposes of this study. Hypotheses were tested using structural equation modeling and the bootstrapping method for mediation analysis. The results showed that ASM and social media usage frequency are distinctive factors in the context of perceived SMMAs. Consumers’ ASM creates a psychological difference that positively affects their perceptions of brands’ SMMAs. Moreover, social media usage frequency negatively moderates the effect of perceived SMMAs on brand equity and purchase intentions. Another important finding is that brand equity partially mediates the impact of perceived SMMAs on purchase intentions. The present article describes the first study to test the effect of consumers’ ASM on perceived SMMAs and to investigate the moderating effect of social media usage frequency regarding the effect of perceived SMMAs on brand equity and purchase intentions. The conceptual framework contains both a mediator and moderator that generated additional insights into the literature regarding the context of this study.

## 1. Introduction

The last decade’s technological advancements reshaped markets and marketing concepts considering consumers and businesses [1]. Social media has been transformed into a crucial phenomenon both for the business world and the research field. In particular, social media, e.g., [2,3,4], and social media marketing, e.g., [5,6,7], topics have been studied in highly regarded journals recently. Today, it is essential for businesses to consider consumers’ psychology and perceptions about social media marketing activities (SMMAs) that create sustainable competitive advantage. Consumers’ perception of SMMAs may create some benefits; however, scant information is available regarding the effect of perceived SMMAs on the creation of brand equity and on consumer behavior towards the brand [8]. Companies are continuously increasing their social media-related marketing budget, but how to obtain benefits from it remains problematic for marketing practitioners.

In the literature, some scholars investigated the effect of perceived SMMAs on brand equity, e.g., [8,9,10]. Other research is concentrated on the effect of perceived SMMAs on the purchase intentions of consumers, e.g., [11,12]. In addition, there are few and contradictory studies regarding the mediating effect of brand equity (or with its dimension) with respect to the impact of perceived SMMAs on purchase intentions, e.g., [5,8,13]. This study contributes to the literature by investigating and presenting findings regarding the contradicting mediating effect of brand equity. Moreover, the role and important components of perceived SMMAs are revealed in the context of brand equity and purchase intentions of consumers, which will help guide marketing practitioners on how to obtain benefits from social media marketing.

To our knowledge, none of the previous researchers incorporated the attachment to social media (ASM) concept (which is a concept that may affect perceived SMMAs) in their studies regarding the effect of perceived SMMAs on brand equity and purchase intentions. As an antecedent of perceived SMMAs, ASM is an important concept since it is closely related to perceptions about SMMAs. An attachment formed with any object may have an effect on perceptions received from that object. As a result, ASM and perceived SMMAs are concepts that are theoretically closely related. The state of consumers’ psychology when receiving marketing communications from brands can be important, and for segmentation and targeting, the degree of consumers’ ASM will provide additional insights if there is an effect on perceived SMMAs. In that sense, there is a gap in the literature. Furthermore, to the best of our knowledge, studies that investigated the effect of perceived SMMAs on brand equity and purchase intentions have not considered the social media usage frequency of consumers as a moderator variable, which may present valuable insights. It is important to note that, as explained in the measurement part of this study, usage frequency is based on the “perceived usage” of the customers. For the rest of this paper, social media usage frequency indicates the perceived usage frequency of social media. Theoretically, studying the effect of perceived SMMAs on brand equity and purchase intentions is important, but it does not differentiate among users. Some consumers use social media more frequently than others, and it is imperative to observe whether usage frequency is an important variable when considering the effect of perceived SMMAs on brand equity and purchase intentions. This will also help marketing practitioners in segmenting and targeting their consumers. This constitutes another research gap in the related research field. To sum up, there are two main unique academic contributions of this study. This study is the first article to investigate the effect of ASM on perceived SMMAs with other related concepts (i.e., brand equity, purchase intentions) that are presented in the conceptual framework. The other main academic contribution is that it is the first study to analyze the moderating effect of social media usage frequency regarding the effect of perceived SMMAs on brand equity and purchase intentions. In that respect, the results enlighten the related research field. Additionally, this paper investigates previously studied relationships in a diverse industry and culture, which makes the findings significant and unique for the extant research. Consequently, the two main research objectives are as follows:

(1) To investigate the effect of consumers’ ASM on their perceived SMMAs;
(2) To observe the moderating effect of consumers’ social media usage frequencies regarding the effect of perceived SMMAs on brand equity and purchase intentions.

In the social media marketing literature, many studies concentrate on different types of industries such as the luxury brands industry, e.g., [8,9], the airline industry, e.g., [14], the automotive industry, e.g., [13], the e-commerce industry, e.g., [12,15] and so on. However, these studies have not investigated the technology industry extensively, which makes the technology industry convenient for the present study. Moreover, the data were collected from Turkish consumers, and there are 57.5 million social media users in Turkey [16]. These make the context of the present study valuable and also add new understanding to the literature, as explained in the Section 3.

This article also makes managerial contributions that are beneficial for marketing practitioners. Understanding the nature and components of perceived SMMAs in the context of brand equity and purchase intentions will help marketing managers in redesigning social media-related marketing communication such as whether to focus on more customized or entertaining, interactive, trendy, and word-of-mouth triggering social media content. Additionally, our findings concerning consumers’ psychological states regarding ASM and social media usage frequencies provide important segmentation and targeting insights, which will enhance marketers’ social media marketing strategies and lead to sustainable competitive advantages.

This paper is organized as follows: The Section 2 presents a review of the extant literature regarding the concepts, conceptual framework, and hypotheses. The Section 3 provides necessary information on the methodology of this study including the sample and procedure/measurement. The Section 4 shows the results of the analyses. In the Section 5, the theoretical contribution of this study and managerial implications are discussed, concluding with limitations and future research directions.

## 2. Conceptual Framework and Hypotheses

This section explains the main concepts regarding the variables in the model followed by the development of the hypotheses. It is important to note that ASM is one of the critical variables in this study that is developed based on attachment theory. Consequently, attachment theory is further explained in this section as the theoretical base of this study, while the remaining theories are only used to justify the specific hypotheses.

### 2.1. Attachment to Social Media

In the marketing literature, the “attachment” concept has been studied in a variety of subjects such as brand attachment/relationships [17,18,19,20], consumer–firm emotional attachment [21], and place attachment [22]. Wang et al. [23] also stated that people can form an attachment to social networking sites. Social networking sites are one of the classifications of social media [24]. In a broader sense, consumer bonding with social media resulted in the concept of ASM. As far as it is known, VanMeter et al. [25] introduced the ASM concept as a holistic approach. Prior to explaining attachment theory and the ASM concept in detail, it is important to explore the root of the concept.

Before the introduction of social media, the internet was the main technological phenomenon that affected the habits and rituals of people. Griffiths [26] pointed out long ago a type of negative bonding with technology (i.e., technological addiction), specifically, computer and internet addiction. Similarly, Kandell [27] studied internet addiction as a pathological use that generates unpleasant feelings for the user. Addiction to social media (or social media addiction) has also been studied in the recent literature, e.g., [28], and with attachment styles such as avoidance and anxiety [29] and secure, insecure–avoidant, insecure–anxious, and disorganized and dismissing [30]. These mostly demonstrate social media’s negative effects on the user’s life, where Altuwairiqi et al. [31] defined it as problematic ASM. On the contrary, Kaposi [32] observed passionate and more positive declarations regarding ASM. Another important topic is whether positive feelings regarding ASM may actually be an addiction and have downsides. However, as per VanMeter et al.’s [25] conceptualization, it is also possible to investigate the previously studied internet/social media addiction (which can be conceptualized as technological addiction) phenomenon from another perspective with a more optimistic ASM concept.

John Bowlby and Mary D. Salter developed attachment theory [33] and defined attachment as “an affectional tie that one person or animal forms between himself and another specific one—a tie that binds them together in space and endures over time” [34] (p. 50). Bowlby [35] (p. 11) conceptualized attachment as “the interactive regulation of synchrony between psychobiologically attuned organisms”. Accordingly, ASM is defined as “the strength of a bond between a person and social media” [25] (p. 71). In their article, ASM is holistically conceptualized, and the related scale is on the individual level. That is, instead of focusing on a single platform such as Facebook or Twitter, consumers’ general ASM is measured. In the literature, other articles [36,37] studied the same concept (attachment to technology) such as perceived online attachment motivation, which is in another context. David and Roberts [38] measured ASM with social media usage intensity, while Helme-Guizon et al. [39] considered ASM as an attachment to Facebook. These approaches contribute to the developing concept of attachment to technology in different contexts. Although it is conceptually connected, ASM also differs from the attitude toward social media, where the latter is mainly related to an individual’s overall feelings about social media [40]. In VanMeter et al.’s [25] paper, ASM consists of eight dimensions, which are connecting, nostalgia, informed, enjoyment, advice, affirmed, enhances my life, and influence.

As social media users feel–live–apply the abovementioned eight concepts by using social media, ASM is formed between individuals and social media. Considering ASM, the present paper started with the eight dimensions of VanMeter et al.’s [25] conceptualization; however, as it is a quite new concept, it should be noted that adaptation of standard dimensionalizations is usually needed for slight adjustments and modifications to the context of this study. Furthermore, rather than investigating an individual’s attachment to a specific social media platform, ASM is considered on the individual level, which measures an individual’s general attachment to the social media concept.

### 2.2. Perceived Social Media Marketing Activities

As consumers’ social media usage increases rapidly, their perceptions of SMMAs become crucial for businesses. Perceived SMMAs have the potential to contribute to branding goals as much as traditional marketing activities or even more [8]. In the literature, Kim and Ko’s [9] conceptualization of perceived SMMAs has been widely used. The root of their conceptualization is the work of Kim and Ko [11]. Their conceptualization consists of five dimensions including entertainment, interaction, trendiness, customization, and word of mouth. Word of mouth is actually a consumer response, and some scholars include it in brand equity or brand loyalty. The present paper considers perceived SMMAs from Kim and Ko’s [9] approach with the abovementioned five dimensions, where word of mouth is one of the dimensions of perceived SMMAs. It is important to note that the conceptualization is about consumers’ perceptions of SMMAs (perceived SMMAs). Kim and Ko’s [9] conceptualization is appropriate for this paper since it has been widely used in the literature and the conceptual model of the present paper consists of constructs that are on the individual level.

Regarding Kim and Ko’s [9] conceptualization, some articles, e.g., [5,8,13,41], used the exact same five dimensions, while other studies applied similar dimensions in common, e.g., [10,42]. This paper began with the five dimensions established in the literature (i.e., entertainment, interaction, trendiness, customization, and word of mouth) while being aware that often standard dimensionalizations need to be adapted, e.g., [9], which is just a modification of the original concept. The construct is based on consumers’ perceptions of brands’ SMMAs.

### 2.3. Brand Equity

There are two different approaches to the brand equity concept in the literature. Some researchers considered brand equity from a financial-based perspective, e.g., [43], while other researchers focused on customer-based brand equity, e.g., [44,45]. A brand’s financial value to a firm is the main focus of financial-based brand equity, whereas a brand’s value to a customer is emphasized in customer-based brand equity [46]. It can be said that financial-based brand equity measures the result of customer-based brand equity [47]. The dominant approach to the brand equity concept in the marketing literature is customer-based. It is important for businesses to build a strong brand with positive brand equity since it increases the probability of actual purchase [10]. Marketers also use brand equity terms implying customers’ perceptions rather than the financial value of the brand [48]. The essence of customer-based brand equity is customers’/consumers’ perceptions. In that sense, the true meaning of financial-based brand valuation can only be understood by studying consumer behavior and by forming an informative vision of a brand in the minds of consumers [45]. This paper also approaches brand equity from a customer-based perspective, and for the rest of this paper, brand equity implies CBBE.

Before the common use of the brand equity term in the literature, Srinivasan [49] indicated brand equity as a brand-specific effect. Farquhar [50] (p. 24) defined brand equity as “the added value with which a given brand endows a product”. Considering the usage intensity of the definitions, Aaker’s [44] and Keller’s [45] definitions of brand equity step forward. Keller [45] (p. 2) defined brand equity as “the differential effect of brand knowledge on consumer response to the marketing of the brand”. As a more comprehensive definition, Aaker [44] (p. 15) defined brand equity as follows:

“A set of brand assets and liabilities linked to a brand, its name and symbol, that add to or subtract from the value provided by a product or service to a firm and/or to that firm’s customers”.

Regarding the dimensionality of brand equity, two of the most generally accepted approaches are those of Aaker [44] and Keller [45]. Keller [45] considered brand equity from a brand knowledge framework as a two-dimensional construct, including brand awareness and brand image. Aaker’s [44] conceptualization of brand equity involves five dimensions including brand awareness, brand associations, brand loyalty, perceived quality, and other proprietary brand assets. However, many of the studies in the literature, e.g., [51,52], exclude the last dimension and consider brand equity as a four-dimensional construct. Some articles combine brand awareness and brand associations into a single dimension, e.g., [53,54]. Nevertheless, as it has been used extensively for the last 20 years, and similar to recent studies, for the brand equity concept, this paper started with the four dimensions of Aaker’s [44] conceptualization including brand awareness, brand associations, brand loyalty, and perceived quality. The results showed that brand awareness and associations merged together, which is in line with previous research, e.g., [55].

Regarding the dimensions of brand equity, Aaker [56] (p. 10) referred to brand awareness as “the strength of a brand’s presence in the consumer’s mind”. It is about the performance of consumers to recall and recognize the brand [45]. Brand awareness has the potential to add value to the brand. Kapferer [57] stated that brand awareness creates a sense of trust for consumers and signals high quality. In addition, awareness has the potential to affect the attitudes and perceptions of consumers [58]. As a result, it increases the probability of brand purchases [59]. Brand associations are also closely related to consumers’ perceptions. Brand association is anything that can be linked or connected in memory to a brand [44]. It includes various kinds of ideas, episodes, and facts linked to a brand [60] that demonstrate consumers’ knowledge regarding the brand [61].

Brand loyalty is related to the attachment of the consumer to the brand [44]. Keller [62] considered brand loyalty as the top component of the brand resonance pyramid. Brands with a brand-loyal customer base are able to create a competitive advantage. Loyal consumers resist switching to another brand and demonstrate more positive responses to a brand compared with non-loyal consumers [60] and trust the brand [63]. Perceived quality is about the quality perceptions of a brand regarding its consumers. Perceived quality can be defined as the consumers’ evaluations of a product’s superiority or excellence [64]. Consequently, perceived quality is related to consumers’ judgments [65]. Since it involves “subjective” perceptions rather than “objective” quality, perceived quality can create important advantages. It creates a reason to purchase and differentiates a brand from its competitors [66].

### 2.4. Purchase Intentions

**Figure 1:**
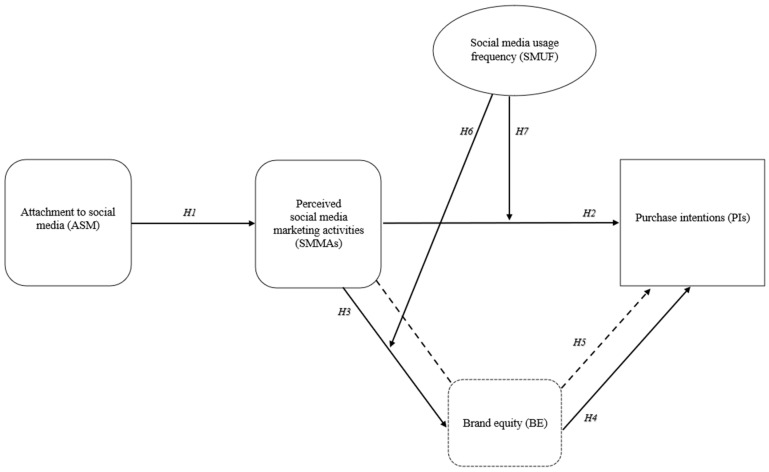
Conceptual framework.

Purchase intentions reflect the possibility that a consumer is planning or willing to buy a product or service in the future [67]. Kim and Ko [9] stated that purchase intention is a combination of consumer interest in a product and the probability of buying the product. Purchase intention is also an essential part of the purchasing process. It is one of the initial stages of the buying process. From a brand management perspective, the purchase intentions concept is related to buying a brand or the probability of switching to another brand [62]. Customer satisfaction may affect purchase intentions [68], which may lead to actual purchases by customers. Summarizing, this paper contains four different concepts including independent, dependent, and mediator variables, as explained above, and one moderator variable, which is solely the social media usage frequency measured as daily social media usage (minutes). For convenience, the conceptual framework of this paper including all variables is shown in Figure 1, which assists in visualizing the next sections that justify the linkages and formal hypotheses.

### 2.5. Direct Effect Hypothesis Development

The ASM concept was developed based on attachment theory. Following Bowlby’s views, attachment theory is based on a child’s bond to his/her mother (target). As one is more strongly attached to a target, one’s proximity to the target increases [18]. A child’s attachment to his/her mother is emotional, long-lasting, and unique. Because of this attachment, the child has strong positive emotional evaluations regarding his/her mother (target) such as “the best cook, the most beautiful person”, etc. Considering the ASM concept, a similar emotional attachment is formed between consumers and social media (target). Perceived SMMAs are communicated to consumers through social media (target). As consumers’ ASM increases, perceived SMMAs may be evaluated more positively by consumers, similar to a child’s positive evaluations of his/her mother’s activities. Recently, the attachment theory perspective has been also considered in the social media context, e.g., [69]. In the social media context, different attachment styles are related to using social media [29]. Drawing on Kandell’s [27] views, the user greatly devotes time and resources as a result of attachment to the internet and, in particular, social media. Attachment to technology may become so salient that the activity may become the most important thing in a person’s life, ruling the person’s thinking and behavior [26]. Additionally, Kaposi [32] reported that users of technology (i.e., internet, smartphones, and social media) consider technology as fundamental as drinking or eating, and they are passionate about their ASM. As a result, consumers’ ASM positively affects perceived SMMAs since social media has become the most prominent aspect that affects their perceptions.

In the literature, some findings also have implications regarding the positive effect of ASM on perceived SMMAs. VanMeter et al. [25] stated that consumers who have stronger ASM form the more desired consumer group regarding marketing initiatives and campaigns. In addition, their study revealed that stronger ASM leads to more positive word-of-mouth (WOM) activities through social media and more social media and consumer behavior-related activities. VanMeter et al. [40] found that consumers who have stronger ASM tend to make WOM recommendations about a brand through social media. Other studies’ results showed that perceived online attachment motivation positively affects online knowledge-sharing behavior [36,37]. These studies did not investigate ASM but rather general online attachment motivation, which is a related concept. Wang et al. [23] found that emotional attachment to social networking sites positively affects users’ social media usage behavior such as posting/sharing on social media. Helme-Guizon et al. [39] concluded that attachment to Facebook increases consumers’ level of activities with a brand on Facebook, where interaction may be inferred. Considering the WOM and interaction dimensions of perceived SMMAs, the results of these studies imply a positive effect of ASM on perceived SMMA.

To sum up, regarding the theoretical base of attachment theory and the literature findings, the first hypothesis of this study is formulated as follows:

**H1.**  *Consumers’ attachment to social media positively affects perceived social media marketing activities.*

Flow theory is an explanatory theory for the positive effect of perceived SMMAs on purchase intentions. Flow is a dynamic state an individual feels that occurs when fully committed to an activity [70]. It is a psychological state when a person is motivated, happy, and cognitively efficient [71]. Flow theory is frequently used in marketing and related areas [72], specifically as an explanatory theory for online consumer behavior and experience [73]. Hoffman and Novak’s [74] study was the first to use flow theory regarding online marketing activities and internet users’ experiences [71]. Cuevas et al. [75] stated that flow is an important concept when designing social media marketing and also found that flow leads to purchase intentions. There is also research on the effect of flow on purchase intentions and/or behavioral intention and similar studies that used flow as an explanatory theory, e.g., [76,77,78,79,80]. Zarei et al. [81] investigated SMMAs and customer response with the moderation of flow theory and found that flow moderates the effect of SMMAs on customer response. This shows that flow is an important concept regarding SMMAs and customer response, and one of the initial stages of customer response can be considered as purchase intentions, which indicates the argumentation of the effect of SMMAs on purchase intentions. Moreover, another study from the online marketing literature found that flow experience contributes to showing more positive sides of the products to customers, leading to higher purchase intentions [82]. Smith and Sivakumar [83] stated that various dimensions of flow can motivate browsing, one-time purchases, and repeat purchase behavior. Consumers have become more engaged with social media, and they are in a flow state when using social media. In the flow state, users are happy and motivated regarding the activity, and Godey et al. [8] found a positive effect of perceived SMMAs on consumers’ responses (including brand preference, which is closely related to purchase intentions). Their study showed that users perceive SMMAs as fun, interesting, and interactive, and these aspects contribute to the flow state of users. As a result, consumers’ perceptions about a brand’s SMMAs are stronger in the flow state and affect their brand preference. Additionally, users also perceive social networking marketing (which is similar to SMMAs) as fun, attractive, interesting, and interactive, which positively affects their purchase intentions [84]. These elements of perceived SMMAs make users’ flow states deeper, contributing to their purchase intentions regarding a brand.

Furthermore, previous research in different contexts such as luxury fashion brands [11], the automotive industry [13], the restaurant industry [85,86], and the e-commerce industry [12] indicated the positive effect of perceived SMMAs on purchase intentions; therefore, the connection to the present conceptual model can be made accordingly. Positive perceptions of SMMAs are transferred to consumers’ purchase intentions regardless of the contextual background, resulting in high conceptual support from the previous literature.

According to the theoretical support from flow theory and the literature findings, the second hypothesis of this study is as follows:

**H2.**  *Perceived social media marketing activities positively affect purchase intentions.*

Brands’ perceived SMMAs are one of the main elements that formulate positive attitudes towards brands and could create higher consumer-based brand equity [87]. Flow theory [70] also supports the explanation of the impact of perceived SMMAs on brand equity. Consumers who are in the flow state perceive brands’ SMMAs more intensely and transfer positive feelings and attitudes to brand equity. Kim and Ko [9] found a positive effect of perceived SMMAs on brand equity, where perceived SMMA elements were mainly composed of entertainment and interaction. Interactivity and fun elements help users to be in the flow state, resulting in a stronger perception of brand equity elements. A user in a flow state perceives SMMAs deeper, which influences his/her perception regarding brand equity. Furthermore, schema theory, introduced by F. Bartlett, is also used by studies, e.g., [15], to explain the effect of perceived SMMAs on brand equity. A schema predicts action when the level of activation is high enough and when the necessary triggering conditions exist [88]. Mainly, schema theory is about previously learned information and the effect of this information on understanding the process. Yadav and Rahman [15] found a positive effect of perceived SMMAs on brand equity based on schema theory, where consumers link communication stimuli with previously formed similar constructs. In that sense, brands’ SMMAs perceived by consumers form information regarding the brand, and that information affects brand equity positively in a pattern.

Although studies on the luxury fashion brand sector [8,9], online education [89], the airline industry [14], the e-commerce industry [12,15], and various product categories [10] have reported a positive effect of perceived SMMAs on brand equity, Ural and Yuksel [13] predicted a positive effect but could not find support for the hypothesis in the automotive industry. There is a conflict in the published findings, and the present paper adds additional insights by providing results regarding a similar cultural context. This constitutes another reason to test and validate the related effect, which is an overall contribution of the present study. Accordingly, the third hypothesis is as follows:

**H3.**  *Perceived social media marketing activities positively affect brand equity.*

Information processing theory explains consumer behavior in the context of cognitive processes [90]. Only one piece of information is needed to choose one of the two similar alternatives [91]. Information processing theory is also used in brand equity-related, e.g., [92], and purchase intention-related, e.g., [93], contexts. Based on information processing theory, a consumer perceives information from outside and then transfers it to memory; when needed, the consumer recalls the necessary information and turns it into consumer behavior. Following the views of Kim and Ko [9], consumers consider using social media to obtain information, and they use this information in their purchase decisions. In line with information processing theory, they reported a positive effect of brand equity on purchase intentions.

As far as we know, Cobb-Walgren et al. [94] were the first to test the effect of brand equity on purchase intentions. Their study revealed a positive effect. Another important study showing the strong relationship between brand equity and purchase intention is described in Washburn and Plank’s [51] paper. Most of the studies related to social media topics in diverse sectors such as luxury fashion brands [8,9], online education [89], and multiple different sectors [95] found a positive effect of brand equity on purchase intentions. Apart from being in different contexts, the conceptualizations of the articles share similarities with the present conceptual framework, providing conceptual support that consumers’ judgments and feelings regarding brand equity-related elements may have an effect on their purchase intentions for a particular brand. Based on the support of information processing theory and the literature findings, the fourth hypothesis of this paper is as follows:

**H4.**  *Brand equity positively affects purchase intentions.*

### 2.6. Mediating and Moderating Effect Hypothesis Development

It is proposed that perceived SMMAs have a positive effect on brand equity and brand equity positively affects purchase intentions. There may be a mediating effect of brand equity. A limited number of studies in the literature investigated the related mediating effect of brand equity. Godey et al. [8] found that brand equity partially mediates the relationship between perceived SMMAs and consumer response. One of the dimensions of consumer response is brand preference, and it can be articulated that brand preference is highly related to purchase intentions. As a result, brand equity could be a mediator between perceived SMMAs and purchase intentions. Coursaris et al. [96] found a mediating effect of brand equity between engaging brand content and purchase intention. Moslehpour et al. [5] found a partial mediating effect of brand image (which is part of brand equity and especially related to brand associations) regarding the effect of social media marketing activities on purchase intentions. Kim and Ko [9] mentioned that perceived SMMAs may enhance purchase intentions through brand equity. On the other hand, Ural and Yuksel [13] hypothesized brand equity’s mediating effect but could not find support. These limited and conflicting results regarding the mediating effect of brand equity suggest that it must be retested. Accordingly, the mediating hypothesis of this paper is as follows:

**H5.**  *Brand equity mediates the effect of perceived social media marketing activities on purchase intentions.*

Some studies consider virtual/online communities or internet users as two distinct groups of posters and lurkers, e.g., [97,98,99]. Similarly, social media users can be classified as posters and lurkers. Posters are users who frequently share/post online messages [100] and product experiences on the internet [97]. In that regard, posters are more experienced and informed users. On the contrary, lurkers rarely post or do not post at all [101]. Consequently, lurkers’ levels of experience are lower compared with posters on social media.

Nonnecke and Preece [101] reported that one of the characteristics of lurkers is that they have limited time. Lurkers have less time available, which is an indicator that posters spend more time on social media. Sun et al. [102] stated that lurkers do not want to spend additional time to maintain a commitment. In the social media context, it can be articulated that lurkers spend less time on social media than posters. In line with this view, Hurtubise et al. [103] reported that the minutes spent on the virtual community of practice (VCoP) for lurkers is less than posters. The present paper measures social media usage frequency as daily minutes spent on social media, and based on the limited literature, it can be stated that consumers who use social media more frequently are posters, while consumers who use social media less frequently are lurkers. As a result, the characteristics of more frequent social media users are similar to posters while lurkers have similar characteristics with less frequent social media users. In that respect, consumers who use social media more frequently are more experienced and well-informed users, so they are affected less regarding perceived SMMAs. More prior information related to a brand is stored in mind for posters than for lurkers. The effect of perceived SMMAs on posters is weak. On the other hand, consumers who use social media less frequently are less experienced consumers, and perceived SMMAs have a higher impact considering brand equity and purchase intentions. In other words, the impact of perceived SMMAs on brand equity and purchase intentions is higher for consumers who use social media less frequently. In a brand-related study, Beukeboom et al. [104] found that time spent on Facebook has a negative and significant relationship with the net promoter score, which is an indicator of brand evaluation. To sum up, social media usage frequency may have a negative moderating effect on the impact of perceived SMMAs on brand equity and purchase intentions. As consumers’ social media usage frequencies increase (daily minutes), the effect of perceived SMMAs on brand equity and purchase intentions may decrease. Accordingly, the last two hypotheses of this study are as follows:

**H6.**  *Social media usage frequency negatively moderates the impact of perceived social media marketing activities on brand equity. Increased social media usage frequency decreases the impact of perceived social media marketing activities on brand equity.*

**H7.**  *Social media usage frequency negatively moderates the impact of perceived social media marketing activities on purchase intentions. Increased social media usage frequency decreases the impact of perceived social media marketing activities on purchase intentions.*

## 3. Methodology

In this section, the methodological aspects of this study such as the procedure, sample, and measurement procedures including exploratory and confirmatory factor analyses are provided.

### 3.1. Sample and Procedure

Two studies (pilot and main) were conducted for the purposes of this research. For both studies, responses were collected on paper in a face-to-face questionnaire format. Brands from the technology industry were selected for this study. Regarding the brands, Apple and Samsung were chosen since they are major technology brands and head-to-head competitors. Additionally, both brands have millions of social media followers and high viewing statistics, which makes these two brands appropriate for this study.

A pilot study was conducted with a sample of 45 respondents in order to check whether the chosen brands and questionnaire design were suitable. They were first asked to pick one of the technology brands that they followed on social media out of 16 international technology brands and answered the questions accordingly. Overall, 38 out of the 45 respondents chose Apple or Samsung, which verified that the two brands are appropriate. In addition, the pilot study was performed face-to-face, and all of the respondents who followed the brand on social media were able to fill out the survey appropriately. The results of the pilot study verified the chosen brands and questionnaire design. In addition, before conducting the main study, the questionnaire obtained official approval from the ethics committee.

Regarding the main study, the sample consists of Turkish youths. Recent research, e.g., [10,54,105], in the context of social media predominantly collected data from students. The context of this paper is highly related to social media usage, and considering the related studies, it was appropriate to collect data from university students. Considering sampling, although we aimed to use simple random sampling, as a result of the convenience of the respondents, only willing and available students participated in the main study. Recent studies in the social media marketing context [42] also considered the convenience of participants regarding sampling. It is also important to mention that the Turkish market is relevant to the context of this study. First of all, similar studies collected data from various countries such as the United States, India, Malaysia, etc., and according to Hosfstede’s Cultural Dimensions, Turkey is quite different than those countries, especially in individualism, uncertainty avoidance, and power distance dimensions [106]. Moreover, there are 57.5 million social media users in Turkey, and considering the internet users, 96% use social media, which is higher than the world’s overall percentage [16]. In addition to this, social media advertising spending in Turkey was estimated to reach approximately USD 380.8m in 2024, and there is a growing trend in this market [107]. Thus, the Turkish market is appropriate and valuable for this study and allowed us to add additional insights both theoretically and managerially. Data were collected face-to-face, where participants chose only one brand (Apple or Samsung) and continued the survey with that particular brand in mind. Following the collection of the raw data, a post-screening procedure was applied. In order to be included in the data analysis, the filled-out questionnaires needed to present the implications of prior information and perceptions of the chosen brands, and respondents needed to perceive (as in the study of Bruhn et al. [108]) any SMMAs of the brand they selected. Any questionnaire with missing and/or inappropriate answers (e.g., not perceiving any SMMAs or not selecting a brand but still filling out the remaining questions) was excluded from this study.

Following post-screening, a total of 907 data points were analyzed. Considering the sample characteristics, 54.2% of the sample was female and 45.8% was male. The majority (65.1%) of the respondents were between 20 and 22 years old. The three most used social media platforms were Instagram, Facebook, and Twitter. Regarding social media usage frequency, which is the moderator variable in this study, the average daily social media usage frequency was 3 h and 12 min.

### 3.2. Measurement

Social media usage frequency was measured by asking the respondents about their daily social media usage (minutes). For this variable, there was no distinction between gaming time and active social media usage time of customers. The respondents only mentioned their daily social media usage minutes based on their perception. Therefore, as stated at the beginning of this paper, this variable reflects the perceived usage frequency of social media. The other four variables were constructs consisting of several items. The ASM scale was adopted from VanMeter et al. [25] since they were the first to develop and validate the holistic ASM scale. The present paper used Kim and Ko’s [9] perceived SMMAs approach, and their scale has been commonly used in the literature. Items for brand equity and purchase intentions were adopted from articles [51,55,109], which have been commonly used in the literature. The items and their references are reported in Appendix A (Table A1). The items were translated into Turkish, and experts consisting of six researchers checked the questionnaire in order to establish face/content validity. A 7-point Likert ranging from “strongly disagree—(1)” to “strongly agree—(7)” was used to measure the items.

Firstly, exploratory factor analysis (EFA) was performed in order to observe the dimensionality of each variable and assess the initial validity of the results. EFA was applied by construct through SPSS software version 17.0 with VARIMAX rotation, which is one of the most commonly used rotation methods [110]. The detailed item–dimension breakdown for each construct can be seen in Table 1, where descriptive statistics of the items are shown.

For each construct, the factor loadings were greater than 0.5 without cross-loadings of the items (>0.5), and the total variance explained (TVE) for each variable was above 60% of the appropriate cutoffs [111]. In addition, Kaiser–Meyer–Olkin (KMO) scores were acceptable as all were greater than 0.6 [39]. Following Nunnally [112], Cronbach’s alpha scores were above 0.7 for every construct, thus establishing their reliability. The detailed results of EFA and the reliability analysis can be seen in Table 2. In summary, the results of EFA were promising for validity and reliability measures. The remaining analyses were conducted with the resulting dimensions and items for each construct.

The hypotheses were tested with structural equation modeling (SEM) using AMOS software version 18.0. It is important to distinguish reflective and formative models regarding SEM. While formative models are not interchangeable, it is possible to remove an item from a reflective model, where the arrows are from the latent variable to the observed variables in the SEM model [113]. Consistent with recent articles, e.g., [114,115], a reflective model was used in this study consisting of reflective–reflective second-order constructs for dimensional variables. A confirmatory factor analysis (CFA) with all latent variables consisting of second-order constructs was performed before testing the hypotheses in order to establish construct validity. Construct validity can be achieved by observing face/content validity (FV), nomological validity (NV), convergent validity (CV), and discriminant validity (DV) [111]. As mentioned before, FV was achieved because experts evaluated the questionnaire. All hypotheses were developed based on theory and/or past research findings that established NV.

As stated above, the CFA model includes second-order constructs corresponding to the results of EFA. Factor loadings, composite reliability (CR), and average variance extracted (AVE) values were used to determine CV. CR values ranged between 0.82 and 0.95, which exceeded the cutoff value of 0.7; AVE values ranged from 0.56 to 0.86, and the factor loadings were all above the acceptable value of 0.5 [111], which achieved CV. The detailed results of CV including factor loadings, AVE, and CR values can be seen in Table 3. Lastly, in order to establish DV, correlations among the constructs had to be below 0.85 [116]. In line with prior research [9], Pearson correlation analysis was performed. The correlations among constructs ranged between 0.51 and 0.80 (Table 4), which were below the acceptable value of 0.85 that established DV. To sum up, the four different validity criteria were met, which resulted in construct validity.

Establishing construct validity is not sufficient to continue with hypothesis testing. Model fit measures are also important. The model yielded an acceptable fit for SEM, which can be seen in Table 5. The CMIN/df value was 2.73, the goodness-of-fit index (GFI) value was 0.88, the adjusted goodness-of-fit index (AGFI) value was 0.87, and the comparative fit index value (CFI) was 0.95, indicating that most of the fit statistics were at or above the acceptable cutoff. In addition, the root mean square error of approximation (RMSEA) value was 0.044, which was below the acceptable value [111,117].

The hypotheses were tested with SEM using AMOS software version 18.0. For the structural model, the construct as a whole approach was used. The conceptualization of the second-order construct is that the sub-dimensions will converge to form the main construct. This idea has been acknowledged by leading scholars, e.g., [118]. Building on this idea, it is logical to aggregate the scores and take their average in an attempt to gauge and test the research, which can be described as “a mediated-moderated structural equation model”. For the moderating effect of social media usage frequency, a moderation analysis was conducted based on Aiken and West’s [119] guidelines. First, the aggregate scores and their average regarding each construct were calculated. Second, the standardized scores (mean scores) for the social media usage frequency and perceived SMMAs were calculated and multiplied to produce the interaction term (SMMAXSMUF). Third, a structural model was constructed, and the “main” effect was also included to prevent a biased estimate of the interaction. This approach has been applied in previous studies, e.g., [120]. The mediating effect of brand equity was tested by performing a bootstrapping method, as performed in recent research, e.g., [121].

## 4. Results

The results are shown in two sub-sections regarding the direct effects and mediating-moderating effects.

### 4.1. Testing the Direct Effect Hypotheses

**Figure 2:**
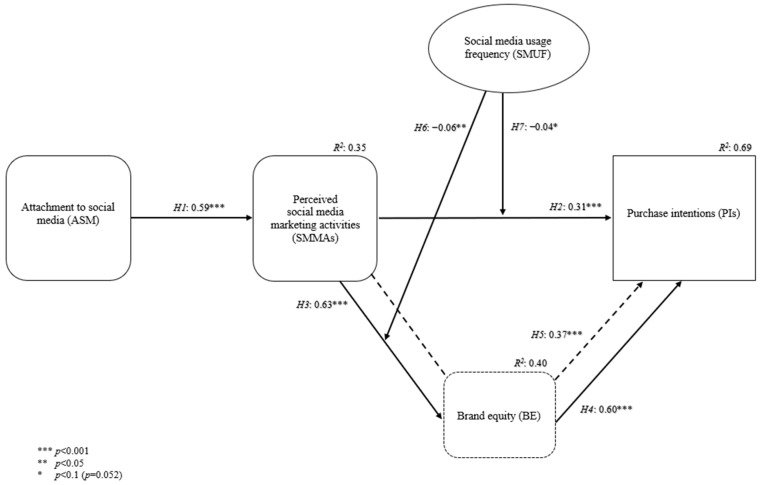
Standardized regression beta coefficients and *R*^2^ values for the conceptual framework.

Figure 2 presents the results of the standardized regression beta coefficients and *R*^2^ values regarding the structural model.

The direct effect hypotheses (H1, H2, H3, and H4) include an independent variable affecting a dependent variable. The results showed that ASM positively affects perceived SMMAs (*β* = 0.59; *t*-value = 22.09; *p* < 0.001) and explains 35% of the variance in perceived SMMAs. Secondly, perceived SMMAs have a positive impact on the purchase intentions of customers (*β* = 0.31; *t*-value = 13.21; *p* < 0.001). The direct impact of perceived SMMAs on purchase intentions has the weakest effect compared with the other direct effect hypotheses. The results showed that perceived SMMAs also positively affect brand equity (*β* = 0.63; *t*-value = 24.36; *p* < 0.001). The effect of perceived SMMAs on brand equity is strongest considering the standardized regression beta coefficients. Perceived SMMAs explain 40% of the variance in brand equity. Lastly, there is a positive effect of brand equity on purchase intentions (*β* = 0.60; *t*-value = 25.05; *p* < 0.001). Regarding the *R*^2^ value of purchase intentions, perceived SMMAs and brand equity explain 69% of the variance in purchase intentions. To conclude, all four direct effect hypotheses (H1, H2, H3, H4) are supported.

### 4.2. Results of Mediating and Moderating Effects

This study utilizes bias-corrected and accelerated (BCa) bootstrap confidence intervals based on 5000 bootstrap runs to assess the significance level of the mediation hypothesis. The bootstrap approach has been shown to outperform Baron and Kenny’s [122] approach [123,124]. Following the guidelines set forth by Zhao et al. [125], results revealed that perceived SMMAs have a complementary indirect effect on purchase intentions through brand equity (β = 0.37; t-value = 16.48; *p* < 0.001) at a 95% confidence interval with the following lower and upper bounds (*p* = 0.001): 0.34–0.41, which that can be seen in Table 6. Thus, H5 received empirical support. It was observed that the indirect effect of perceived SMMAs on purchase intentions is a little larger than the direct effect, which accounts for a lot of BE’s impact on PIs.

Regarding the moderation hypotheses, H6 and H7 are supported, but the effects are weak. The results showed that social media usage frequency negatively moderates the effect of perceived SMMAs on brand equity, supporting H6 (*β* = −0.06; *t*-value = −2.45; *p* < 0.05). As consumers use social media more frequently, the impact of perceived SMMAs on brand equity decreases. Similarly, social media usage frequency acts as a negative moderator regarding the impact of perceived SMMAs on purchase intentions (*β* = −0.04; *t*-value = −1.94; *p* = 0.052), which is qualified as marginally significant and supports *H7*. Increased social media usage frequency decreases the effect of perceived SMMAs on purchase intentions. Table 7 presents the results of all hypotheses in this study.

In conclusion, all seven hypotheses developed in this study are supported with considerable direct effects, partial mediation, and weak negative moderation results.

## 5. Discussion and Conclusions

The Section 5 consists of three sub-sections describing our theoretical contributions, the managerial implications of the results, and the limitations of this study and future research directions.

### 5.1. Theoretical Contribution

In line with previous research on other industries such as the luxury brand sector [8,9], the airline industry [14], online education [89], the e-commerce industry [12,15], and various product categories [10], the positive effect of perceived SMMAs on brand equity was found on the technology industry. The argument for this positive effect can be inferred by both flow theory [70] and schema theory, as described by F. Bartlett. The favorable psychological condition created by the flow state while using social media makes consumers’ perceptions of SMMAs more positive, translating into higher perceptions of brand equity. Moreover, in line with schema theory, the acquired information from SMMAs affects the understanding process of consumers regarding brand equity in a positive manner. However, Ural and Yuksel [13] did not observe a similar effect on the automotive industry in a similar cultural context. It is an important theoretical contribution to observe a related effect that could not be found in a study within a similar cultural context. One of the reasons why the effect could not be observed in the automotive industry but was found in the technology industry may be because of the intensity of technology-related social media marketing actions from various brands compared with automotive brands. According to Interbrand’s Best Global Brands 2023 list [126], the top five brands are all technology-related brands, which may explain the reasoning since they are the most valuable brands as they invest heavily in marketing programs. In summary, the present study validates the majority of the past research findings in a different industry and cultural context. In addition, the results showed the positive effect of perceived SMMAs on purchase intentions, as described in previous research [11,12]. Similar to the above reasoning, there is a shift towards top-valued brands to technology-related brands and, as observed in other industries, our results suggest that consumers perceive diverse marketing activities through social media, which affects their psychological process regarding deciding to buy a product or service in a favorable way, creating an intention to purchase from the brand. Moreover, the reasoning for this effect can also be inferred by flow theory [70] because consumers who are in the flow state when using social media are in a positive psychological mood that affects their perception of SMMAs in a much more favorable way regarding their intentions to purchase. As generally reported in past research [8,9,94], the present paper reported a positive effect of brand equity on purchase intentions. Based on the information processing theory proposed by G. A. Miller, the brand-related information stored in the memory of consumers is recalled when needed in a buying situation, positively affecting purchase intentions. Consistent with previous research, brand awareness and associations merged and formed a single factor in the present study. Together with all dimensions, consumers who recognize or recall a brand easily have positive associations and images of the brand, perceive good quality, become loyal to the brand, and have more intention to purchase the brand because of the positive perceptions created by brand equity. In line with Kim and Ko’s [9] statement, the perceived SMMAs impact purchase intentions through brand equity. There are also contradictory results regarding the mediating effect in the literature. Godey et al. [8] found a mediating effect, and Moslehpour et al. [5] reported a partial mediating effect of a brand’s image regarding the effect of SMMAs on purchase intentions. However, Ural and Yuksel [13] could not find support for the mediation hypothesis. The results showed partial mediation of brand equity, which makes valuable theoretical contributions in that regard. This logic can be again based on the abovementioned fact that technology-related brands are on the rise in the market, with high levels of brand building and marketing communication efforts through social media that have an effect first on brand equity and then on the purchase intentions of customers. However, brand equity is only a partial mediator because the power of SMMAs is intense enough to create its own effect on purchase intentions, especially for the younger generations who use social media frequently.

One of the main results of this study is that ASM positively affects perceived SMMAs. Day to day, consumers use social media more frequently and bond with social media. Yet, the attachment levels differ, which has important implications. Drawing on attachment theory [33], the findings showed that consumers’ psychological states change as their ASM increases, which has a positive impact on their perceptions of SMMAs. This finding can also be explained by various views. Because of an attachment to the internet, a user dedicates considerable resources and time [27], which is also true for social media. Furthermore, Griffiths [26] pointed out that a user may become heavily attached to technology, which may guide user behavior and thinking. Moreover, Kaposi [32] concluded that the internet, smartphones, and social media users are passionate about their ASM and believe that technology is a fundamental need such as eating and drinking. Consequently, consumers who have high ASM consider social media as a core fundamental need in life that affects their psychology and perceptions of SMMAs in a more positive manner. As a theoretical contribution, the present article describes the first study to incorporate the ASM concept regarding the relationships among perceived SMMAs, brand equity, and purchase intentions. As recent research considers the attachment theory perspective in similar contexts, e.g., [69], the results of the present study are highly relevant and enlighten the literature significantly.

The second most important result of the present study is related to the negative moderating effect of social media usage frequency. Recent research, i.e., [127], also considered a similar moderator (i.e., social media usage intensity) in a social media marketing-related topic. As stated before, the impact of perceived SMMAs on brand equity and purchase intentions has been studied in the literature. However, consumers use social media with divergent frequencies. As a result, an important theoretical contribution can be made by testing the related relationships while taking consumers’ social media usage frequency into account. It can be expected that the moderating effect is positive. That is, as consumers’ social media usage frequency increases, they will be more exposed to SMMAs that will positively moderate the impact of perceived SMMAs on brand equity and purchase intentions. However, based on poster–lurker categorization [97], it was hypothesized that social media usage frequency negatively moderates the impact of perceived SMMAs on brand equity and purchase intentions. Accordingly, a consumer who uses social media more frequently has high knowledge and experience with a brand, so the effect of perceived SMMAs is lower. In other words, as consumers’ social media usage frequency increases, they become less sensitive to SMMAs and do not “fall for” the related marketing activities. Support for negative moderating effects was found but with weak effects. Nonnecke and Preece [101] reported that lurkers have less time available; Sun et al. [102] mentioned that lurkers are not willing to spend extra time, and Hurtubise et al. [103] found that lurkers spent less time on virtual community of practice (VCoP) than posters. Therefore, it can be inferred that consumers who spend less time on social media are lurkers who are less knowledgeable and experienced consumers, and consequently, they are much more influenced by SMMAs, which explains the reasoning behind the negative moderating effects. Although the effects were weak, theoretically, they make an important contribution since this is the first study to incorporate social media usage frequency as a moderator variable regarding the impact of perceived SMMAs on brand equity and purchase intentions. The weak effects may be due to the measurement of the social media usage frequency variable. As mentioned before, this variable is based on the “perception” of the customers, not the actual time spent on social media. Because of this, a respondent who reported 4 h of social media usage may actually spend 2 h on social media solely. This may change the strength of the related effect. Moreover, the weak effect means that other moderating variables such as engagement might better explain the relationships. Analyzing only the usage frequency may not show the most important factor in this model as a moderator. Lastly, the SMMA levels of social media platforms vary considerably. A customer spending most of her/his time on WhatsApp compared with Instagram will be exposed to fewer SMMAs, which is not considered in this research. This may also explain the weak moderating effects of usage frequency.

To conclude, the present study revealed that ASM and social media usage frequency are distinct factors in the context of perceived SMMAs. VanMeter et al. [40] also emphasized that a consumer’s ASM drives more meaningful social media interactions with brands and organizations compared with time spent on social media (social media usage frequency). This also shows the diverse effects of ASM and social media usage frequency. Consumers’ ASM has a positive effect on their perceptions of SMMAs. However, as consumers use social media more frequently, the effect of perceived SMMAs on brand equity and purchase intentions decreases.

### 5.2. Managerial Implications

The results of the present study showed that the first dimension of perceived SMMAs includes elements from trendiness, interaction, and entertainment, while the second dimension is about WOM. In that regard, some recommendations may be inferred based on the specific elements of perceived SMMAs that lead to a sustainable competitive advantage. Marketers should design social media content that is perceived by consumers as more fun, interesting, and trendy in order to create a positive effect regarding brand equity and purchase intentions both for B2C and B2B contexts. Based on Zhang and Du’s [128] findings, consumers care more about emotional content in B2C compared with B2B activities. Moreover, as social media contents trigger more WOM and interactions, the impact of perceived SMMAs on brand equity and purchase intentions increases. Interactivity was also found to be an important component in the B2B context, e.g., [128]; so, for both B2B- and B2C-focused companies, marketing practitioners need to set some key communication objectives, such as creating imagery and personality, inspiring action, connecting people, and eliciting emotions [1], and convey a consistent message including elements from perceived SMMAs. The results indicated that the influence of perceived SMMAs on brand equity is stronger compared with purchase intentions. Marketing practitioners can use SMMAs more for brand building and as a call-to-action tool. Considering the AIDA model, the perceptions regarding the related SMMAs will trigger the attention, interest, and desire of consumers and result in action. Particularly, brands that are in the early stage of brand building can benefit from SMMAs as a cost-effective and highly efficient marketing medium. Moreover, new product development (NPD) managers can utilize SMMAs (as consumers perceive SMMAs) to inform consumers and stimulate primary demand for new products that are in the introduction stage of the product life cycle.

Regarding the positive effect of brand equity on purchase intentions, in the social media context, a communication message may include elements that create awareness backed with positive associations. Additionally, enhancing loyalty and focusing on perceived quality aspects of brand equity is important. Building and strengthening loyalty and perceived quality through social media may be more effective considering the instant communication and targeted profile aspects of social media. Considering the partial mediating effect of brand equity, in order to increase the purchase intentions of customers and sales eventually, perceived SMMAs and brand equity concepts should be taken into consideration together for better marketing strategies. It is important for managers to design IMC carefully and deliver a consistent message from each channel that builds the brand, leading to higher purchase intentions.

Companies are starting to invest in digital media more as a form of marketing communication and brand-building tool. In that respect, in order to create an effective and efficient strategy, marketers should make careful segmentation and target consumers who have stronger ASM. This logic is also in line with previous research that stated consumers who have stronger ASM form a more desirable consumer group regarding marketing initiatives and campaigns [25]. Theoretically, perceived SMMAs positively affect brand equity and purchase intentions; and as an antecedent of perceived SMMAs, the ASM concept should be taken into consideration by marketing managers. Targeting consumers who have stronger ASM will result in higher perceived SMMAs, which in turn positively influence brand equity and purchase intentions. In order to find out the related segment, marketers could design and invest in promotional social media content including a survey regarding consumers’ ASM. These may be useful to locate consumers who are more psychologically ready to receive marketing communications, resulting in positive online consumer behavior.

Lastly, the results indicated a negative moderating effect of social media usage frequency. Although the negative moderating effect is weak, it is still important for marketing practitioners since the social media marketing concept is quite new for social media marketing managers and is sometimes not considered a part of integrated marketing communications efforts [129]. First of all, marketers should make careful segmentation in the social media context. Targeting consumers who use social media less frequently is an important marketing strategy to increase the impact of perceived SMMAs. Less frequent social media users will perceive and be influenced by SMMAs more, which contributes to brand equity and purchase intentions. Considering ASM and SMUF together, marketers could pursue a selective specialization strategy regarding market targeting. Marketing practitioners should carefully analyze different social media platforms in order to reveal the potential segments based on consumers’ ASM and their usage frequencies. Accordingly, targeting these two segments in specific social media platforms will result in greater benefits, which will ensure a more sustainable competitive advantage for firms.

### 5.3. Limitations and Future Research Directions

Apart from the theoretical and managerial contributions made to the literature, the present study has some limitations that open an avenue for future research. To begin with, scant information is available considering the effect of ASM on perceived SMMAs and the negative moderating effect of social media usage frequency regarding the effect of perceived SMMAs on brand equity and purchase intentions. Future research should retest these effects in different cultural contexts and/or industries to constitute a meaningful and valid theoretical base for these relationships. Additionally, it is also important to test a similar model with other significant variables, such as consumer engagement, trust, and social influence, which can be investigated in upcoming studies. Data were collected from Turkish university-level youths. Future research may apply a similar conceptual framework in other cultural contexts and different consumer groups. Two brands from the technology industry were selected for the purposes of this study, which may also have limitations. The selected brands are popular brands with loyal customer bases, which may have an effect on the generalizability of the results and hinder observing the real effects of SMMAs. The conceptual model may be tested in technology or other industries with moderate brands and less “fanatic” fans as customers in future studies. It is also important to note that different social media platforms have diverse interactivity and SMMA levels that may affect consumers’ attachment to social media and usage frequencies, which may alter the effect of the other variables in the model. Future studies should test the related model on specific and different social media platforms. Moreover, social media usage frequency was measured solely by asking respondents to state their daily social media usage in minutes without distinguishing their real active time on social media and their gaming, shopping time, etc., on the internet. Because of this, usage frequency reflected only the respondents’ perceived usage frequencies, not the actual time spent on social media, which may have an effect on the results. Future studies should measure the actual time spent on different social media platforms (such as looking at Instagram’s settings menu for the exact usage for the last month’s daily average, etc.) and retest a similar model. It was assumed that consumers who use social media more frequently are posters, while consumers using social media less frequently are lurkers. Future research may investigate whether posters use social media more frequently and whether lurkers use social media less frequently. Lastly, the negative moderating effect of social media usage frequency was present but weak, which may have other explanations. Future studies may consider social media usage frequency as a moderator with other significant and related variables to uncover the strong moderation effects and meanings of usage frequency.

## Figures and Tables

**Table 1:** Descriptive statistics of the items.

Construct	Dimension(s)	Item	Mean	Std. Deviation	Variance
Attachment to social media	Influence, advice, and affirmed	INFL1	3.78	2.00	4.02
		INFL2	3.55	2.08	4.32
		INFL3	3.75	2.04	4.18
		INFL4	4.05	1.99	3.94
		INFL5	4.17	1.96	3.84
		ADV2	4.17	1.92	3.69
		ADV3	4.22	1.89	3.57
		AFF2	4.08	1.98	3.90
	Connecting	CON1	5.71	1.49	2.22
		CON2	6.01	1.26	1.59
		CON3	5.72	1.38	1.90
		CON4	5.74	1.39	1.92
	Informed	INF1	5.52	1.46	2.14
		INF2	5.77	1.33	1.76
		INF3	5.52	1.53	2.33
	Enjoyment	ENJ1	5.24	1.56	2.42
		ENJ2	5.32	1.54	2.36
		ENJ3	5.36	1.45	2.11
	Nostalgia	NOS1	5.62	1.44	2.08
		NOS2	5.28	1.60	2.57
		NOS3	5.44	1.54	2.37
Perceived social media marketing activities	Trendiness, interaction, and entertainment	TRE1	5.34	1.39	1.94
		TRE2	5.69	1.40	1.97
		INT1	4.98	1.55	2.41
		INT2	5.09	1.56	2.42
		INT3	5.17	1.54	2.38
		ENT2	4.93	1.51	2.28
	Word of mouth	WOM1	4.60	1.72	2.98
		WOM2	4.03	1.93	3.74
Brand equity	Brand awareness and associations	BAW1	6.17	1.22	1.49
		BAW2	6.28	1.14	1.30
		BAW3	6.30	1.14	1.30
		BAS1	6.09	1.24	1.53
		BAS2	6.37	1.18	1.38
		BAS3	6.31	1.14	1.29
	Brand loyalty	BLO1	4.69	1.89	3.59
		BLO2	5.25	1.80	3.26
		BLO3	4.69	1.98	3.90
	Perceived quality	PQ1	5.65	1.47	2.16
		PQ2	5.49	1.54	2.37
		PQ3	5.70	1.35	1.82
Purchase intentions	(Single factor)	PI1	5.48	1.62	2.63
		PI2	5.41	1.66	2.75
		PI3	5.38	1.69	2.87

**Table 2:** Exploratory factor analysis (EFA) and reliability analysis (Cronbach’s alpha scores) results.

Construct	Dimension(s)	Item	Factor Loadings	Cronbach’s Alpha	TVE	KMO
Attachment to social media	Influence, advice, and affirmed	INFL1	0.84	0.93	75.8%	0.931
		INFL2	0.89			
		INFL3	0.87			
		INFL4	0.82			
		INFL5	0.79			
		ADV2	0.61			
		ADV3	0.65			
		AFF2	0.72			
	Connecting	CON1	0.83			
		CON2	0.81			
		CON3	0.83			
		CON4	0.79			
	Informed	INF1	0.78			
		INF2	0.81			
		INF3	0.82			
	Enjoyment	ENJ1	0.81			
		ENJ2	0.81			
		ENJ3	0.70			
	Nostalgia	NOS1	0.69			
		NOS2	0.84			
		NOS3	0.80			
Perceived social media marketing activities	Trendiness, interaction, and entertainment	TRE1	0.81	0.87	65.8%	0.860
		TRE2	0.82			
		INT1	0.64			
		INT2	0.67			
		INT3	0.73			
		ENT2	0.61			
	Word of mouth	WOM1	0.78			
		WOM2	0.87			
Brand equity	Brand awareness and associations	BAW1	0.81	0.91	78.8%	0.906
		BAW2	0.88			
		BAW3	0.88			
		BAS1	0.76			
		BAS2	0.82			
		BAS3	0.82			
	Brand loyalty	BLO1	0.89			
		BLO2	0.76			
		BLO3	0.83			
	Perceived quality	PQ1	0.80			
		PQ2	0.83			
		PQ3	0.77			
Purchase intentions	(Single factor)	PI1	0.95	0.95	90.6%	0.769
		PI2	0.96			
		PI3	0.95			

**Table 3:** Convergent validity (CV) results.

Construct	Dimension(s)	Item	Factor Loadings	CR	AVE
Attachment to social media	Influence, advice, and affirmed	INFL1	0.85	0.86	0.56
		INFL2	0.84		
		INFL3	0.85		
		INFL4	0.85		
		INFL5	0.82		
		ADV2	0.58		
		ADV3	0.64		
		AFF2	0.70		
	Connecting	CON1	0.78		
		CON2	0.83		
		CON3	0.90		
		CON4	0.88		
	Informed	INF1	0.80		
		INF2	0.91		
		INF3	0.84		
	Enjoyment	ENJ1	0.85		
		ENJ2	0.85		
		ENJ3	0.81		
	Nostalgia	NOS1	0.81		
		NOS2	0.90		
		NOS3	0.90		
Perceived social media marketing activities	Trendiness, interaction, and entertainment	TRE1	0.69	0.84	0.72
		TRE2	0.69		
		INT1	0.69		
		INT2	0.63		
		INT3	0.70		
		ENT2	0.75		
	Word of mouth	WOM1	0.84		
		WOM2	0.66		
Brand equity	Brand awareness and associations	BAW1	0.83	0.82	0.62
		BAW2	0.91		
		BAW3	0.90		
		BAS1	0.74		
		BAS2	0.75		
		BAS3	0.76		
	Brand loyalty	BLO1	0.77		
		BLO2	0.91		
		BLO3	0.87		
	Perceived quality	PQ1	0.94		
		PQ2	0.91		
		PQ3	0.78		
Purchase intentions	(Single factor)	PI1	0.93	0.95	0.86
		PI2	0.94		
		PI3	0.91		

**Table 4:** Correlation matrix among constructs.

	Mean	Std. Deviation	ASM	Perceived SMMAs	BE	PIs
ASM	4.96	1.10	1			
Perceived SMMAs	4.98	1.14	0.59 **	1		
BE	5.75	1.04	0.51 **	0.64 **	1	
PIs	5.42	1.58	0.53 **	0.70 **	0.80 **	1

** *p* < 0.01.

**Table 5:** Measurement model indices.

Index	Result
Chi/df (CMIN/df)	2.73
GFI	0.88
AGFI	0.87
CFI	0.95
RMSEA	0.044

**Table 6:** Results of the mediation hypothesis test.

Hypothesis	Direct Effect (β)	Direct Effect (t-Value)	Indirect Effect (β)	Indirect Effect (*t*-Value)	Lower Bound	Upper Bound	Result
H5. Perceived SMMAs→BE→PIs	0.31 ***	13.21	0.37 ***	16.48	0.34	0.41	Supported (Partial mediation)

*** *p* < 0.001.

**Table 7:** Comprehensive results of the structural model.

Hypothesis	β	*t*-Value	Result
H1. ASM→Perceived SMMAs	0.59 ***	22.09	Supported
H2. Perceived SMMAs→PIs	0.31 ***	13.21	Supported
H3. Perceived SMMAs→BE	0.63 ***	24.36	Supported
H4. BE→PIs	0.60 ***	25.05	Supported
H5. Perceived SMMAs→ BE→PIs	0.37 ***	16.48	Supported
H6. SMMAxSMUF (-) moderation on perceived SMMAs→BE	−0.06 **	−2.45	Supported
H7. SMMAxSMUF (-) moderation on perceived SMMAs→PIs	−0.04 *	−1.94	Supported

*** *p* < 0.001, ** *p* < 0.05, * *p* < 0.1 (*p* = 0.052).

## Data Availability

The data are available on request from the corresponding author.

## Appendix A

**Table A1:** Items of the constructs with references.

Construct	Item	Reference(s)
Attachment to social media Dimensions: Connecting Nostalgia Informed Enjoyment Advice Affirmed Enhances my life Influence	CON1: “I use social media to interact with friends”.	VanMeter et al. [25]
	CON2: “Social media provides a way for me to stay connected to people across distances”.	
	CON3: “I use social media because it makes staying in touch with others convenient”.	
	CON4: “Social media provides a way for me to keep in touch with others that I care about”.	
	NOS1: “Social media allows me to look back at meaningful events, people, and places from my past”.	
	NOS2: “Using social media makes me feel nostalgic about things that I have done in the past”.	
	NOS3: “Sometimes social media reminds me of warm memories from my past”.	
	INF1: “Social media is one of the main ways I get information about major events”.	
	INF2: “Social media allows me to stay informed about events and news”.	
	INF3: “Social media is one of my primary sources of information about news”.	
	ENJ1: “I use social media as a way for me to de-stress after a long day”.	
	ENJ2: “I use social media to give myself a break when I’ve been busy”.	
	ENJ3: “Social media is an enjoyable way to spend time”.	
	ADV1: “I seek advice for upcoming decisions using social media”.	
	ADV2: “I get advice about medical questions on social media”.	
	ADV3: “If I’m unsure about an upcoming decision I get input from friends on social media”.	
	AFF1: “When others comment on my posts I feel affirmed”.	
	AFF2: “When people respond to my posts in social media I feel like they care about me”.	
	AFF3: “It makes me feel accepted when people comment on my social media posts”.	
	EML1: “Social media makes my life a little bit better”.	
	EML2: “Social media enhances my life”.	
	EML3: “My life is a little richer because of social media”.	
	INFL1: “Sometimes I post things just to have a positive effect on other peoples’ moods”.	
	INFL2: “I post on social media to brighten other peoples’ day”.	
	INFL3: “I post things on social media that I think will be helpful to my friends’ lives”.	
	INFL4: “I want to inspire other people with my social media posts”.	
	INFL5: “I think it is important to share things on social media so those I care about stay informed”.	
Perceived social media marketing activities Dimensions: Entertainment Interaction Trendiness Customization Word-of-mouth	ENT1: “Using brand X’s social media is fun”.	Kim and Ko [9]
	ENT2: “Contents shown in brand X’s social media seem interesting”.	
	INT1: “Brand X’s social media enables information sharing with others”.	
	INT2: “Conversation or opinion exchange with others is possible through brand X’s social media”.	
	INT3: “It is easy to deliver my opinion through brand X’s social media”.	
	TRE1: “Contents shown in brand X’s social media is the newest information”.	
	TRE2: “Using brand X’s social media is very trendy”.	
	CUS1: “Brand X’s social media offers customized information search”.	
	CUS2: “Brand X’s social media provides customized service”.	
	WOM1: “I would like to pass along information on brand, product, or services from brand X’s social media to my friends”.	
	WOM2: “I would like to upload contents from brand X’s social media on my blog or micro blog”.	
Brand equity Dimensions: Brand awareness Brand associations Brand loyalty Perceived quality	BAW1: “I can recognize brand X among other competing brands”.	Yoo and Donthu [55] Washburn and Plank [51]
	BAW2: “I am aware of brand X”.	
	BAW3: “I know what brand X looks like”.	
	BAS1: “Some characteristics of brand X come to my mind quickly”.	
	BAS2: “I can quickly recall the symbol or logo of brand X”.	
	BAS3: “I can easily imagine brand X in my mind”.	
	BLO1: “I consider myself to be loyal to brand X”.	
	BLO2: “Brand X would be my first choice”.	
	BLO3: “I will not buy other brands if brand X is available at the store”.	
	PQ1: “The likely quality of brand X is extremely high”.	
	PQ2: “The likelihood that brand X would be functional is very high”.	
	PQ3: “Brand X is of high quality”.	
Purchase intentions	PI1: “I would like to buy brand X”.	Yoo and Donthu [55] Chen and Chang [109]
	PI2: “I intend to purchase brand X”.	
	PI3: “I am willing to purchase brand X in the future”.	
